# Influence of MMR, MGMT Promotor Methylation and Protein Expression on Overall and Progression-Free Survival in Primary Glioblastoma Patients Treated with Temozolomide

**DOI:** 10.3390/ijms24076184

**Published:** 2023-03-24

**Authors:** Konstantin R. Brawanski, Susanne Sprung, Christian F. Freyschlag, Romana Hoeftberger, Thomas Ströbel, Johannes Haybaeck, Claudius Thomé, Claudia Manzl, Anna M. Birkl-Toeglhofer

**Affiliations:** 1 Department of Neurosurgery, Medical University of Innsbruck, 6020 Innsbruck, Austria; 2 Institute of Pathology, Neuropathology and Molecular Pathology, Medical University of Innsbruck, 6020 Innsbruck, Austria; 3 Division of Neuropathology and Neurochemistry, Department of Neurology, Medical University of Vienna, 1090 Vienna, Austria; 4 Diagnostic and Research Center for Molecular Biomedicine, Diagnostic and Research Institute of Pathology, Medical University of Graz, 8036 Graz, Austria

**Keywords:** glioblastoma, *MGMT* promoter methylation, MGMT protein expression, mismatch repair, temozolomide, immunohistochemical analysis

## Abstract

Glioblastoma is the most common malignant brain tumor in adults. Standard treatment includes tumor resection, radio-chemotherapy and adjuvant chemotherapy with temozolomide (TMZ). TMZ methylates DNA, whereas O6-methylguanine DNA methyltransferase (MGMT) counteracts TMZ effects by removing the intended proteasomal degradation signal. Non-functional MGMT mediates the mismatch repair (MMR) system, leading to apoptosis after futile repair attempts. This study investigated the associations between *MGMT* promoter methylation, MGMT and MMR protein expression, and their effect on overall survival (OS) and progression-free survival (PFS) in patients with glioblastoma. *MGMT* promoter methylation was assessed in 42 treatment-naïve patients with glioblastoma WHO grade IV by pyrosequencing. MGMT and MMR protein expression was analyzed using immunohistochemistry. *MGMT* promoter methylation was present in 52%, whereas patients <70 years of age revealed a significantly longer OS using a log-rank test and a significance threshold of *p* ≤ 0.05. MGMT protein expression and methylation status showed no correlation. MMR protein expression was present in all patients independent of MGMT status and did not influence OS and PFS. Overall, *MGMT* promoter methylation implicates an improved OS in patients with glioblastoma aged <70 years. In the elderly, the extent of surgery has an impact on OS rather than the *MGMT* promoter methylation or protein expression.

## 1. Introduction

Despite advances in surgery, radiation and chemotherapy, the diagnosis of glioblastoma multiforme (GBM) is devastating and the outcome poor. Next to neurological examination, diagnostic tools, i.e., magnetic resonance imaging (MRI), computed tomography [1] and classical histopathology, as well as other invasive and advanced methods [2,3], provide the basis for histopathological diagnosis of GBM. Tumor resection might be a curative intervention in the case of a gross total resection (GTR) compared to a subtotal resection (STR). GTR is defined as the complete removal of all tumor tissue, supported by imaging approaches. In addition to the surgical intervention, treatment including temozolomide (TMZ)-based chemoradiotherapy is used as a standard of care for GBM [4]. Many studies showed that O6-methylguanin DNA methyltransferase (*MGMT*) promoter methylation serves as a prognostic factor for progression-free survival (PFS) and overall survival (OS) associated with a positive response to alkylating agents, such as TMZ, in newly diagnosed GBM patients [5,6,7,8,9,10]. On the contrary, low levels of *MGMT* promoter methylation are associated with an increased resistance against alkylating agents [6,11,12]. Controversially discussed is the MGMT protein expression as a predictive marker for the TMZ response in GBM patients. Spiegl-Kreinecker et al., considered MGMT protein expression superiorly predictive than its promoter methylation [8]. In contrast, Uno et al., indicated that *MGMT* promoter methylation is more reliable to adjuvant therapy and prognosis than MGMT protein expression [13].

With respect to the current standard therapy, patients below the age of 70 years receive resection, followed by concomitant radio-chemotherapy and subsequent adjuvant chemotherapy with TMZ, independent of MGMT promoter methylation [14]. Independent of *MGMT* promoter methylation, OS is 20.5 months in newly diagnosed GBM with tumor-treating fields in addition to current standard therapy [15]. In the elderly (age >70 years), treatment depends on the Karnofsky performance score (KPS) and *MGMT* promoter methylation status. Thus, elderly patients with a low KPS and a verified *MGMT* promoter methylation receive solely chemotherapy with TMZ, while patients with an absent *MGMT* promoter methylation rather receive radiation therapy [16,17].

TMZ alkylates the DNA at different positions, generating pre-apoptotic DNA lesions, whereas only 10% of cytotoxic lesions triggered by O6-methylguanin (O6-MG) are directly attributable to TMZ [18]. In general, mammalian cells have different mechanisms to counteract induced DNA damage by DNA-damage response (DDR) systems, such as the DNA mismatch repair (MMR) system, or base excision repair (BER). The cytotoxic effect of TMZ is based on an impaired interaction of MGMT and the MMR system [6,12,19]. MGMT is a cellular DNA repair protein, which typically removes the methylation of O6-MG and, hence, neutralizes the cytotoxic effect of TMZ [20,21]. Functional MGMT blocks the tumor cell death mediated by alkylating drugs such as TMZ. The epigenetic dysregulation of MGMT transcription leads to *MGMT* promotor methylation and inhibition of its transcription. In this scenario, MGMT is silenced and the O6-MG, which is paired with thymine, is not neutralized by MGMT during DNA replication, the mismatched base pair (O6-MG with thymine) is recognized by the MMR system [22]. With the attempt of correction by MMR, the newly synthesized daughter strand is replaced instead of O6-MG and the mismatch persist. During the next DNA replication, the MMR system aims to fix the mismatch. This leads to a “futile-repair cycle” generating single- and double-strand breaks in the DNA [19,23], causing apoptosis and cell death [24,25,26]. Interestingly, a recent study reported that the apoptosis-inducing factor protein (AIF) behaves like an apoptotic nuclease under increased Ca^2+^ levels [27]. Under alkylating conditions, the AIF-containing DNA–degradosome complex degrades DNA [28]. However, alterations in the MMR system, e.g., deletion of one of the key players, such as MLH1, causes microsatellite instability and is associated with several cancers [29,30,31]. Findings of The Cancer Genome Atlas showed a differing mutation rate between treated and untreated GBMs [32]. Several studies demonstrated alterations of mismatch repair genes (MLH1, MSH2, MSH6, PMS2) in recurrent GBM after TMZ treatment [32,33,34,35].

The aim of this study was to clarify the influence of *MGMT* promoter methylation, MGMT protein expression, as well as the expression of the MMR, and the impact of the extent of surgery on OS and PFS in primary-diagnosed GBM patients.

## 2. Results

### 2.1. Patient Characteristics

Basic characteristics of 42 analyzed GBM patients are summarized in Table 1. The median age of the patients was 67 years at the time of surgery, with a range of 58.0–74.8 years and a sex distribution of 16f:26m (1:1.6). Stratification by age into younger patients (<70 years) and elderly patients (≥70 years) included 23 (54.8%) and 19 (45.2%) individuals, respectively. The mean KPS was 86.3 (±15.45 SD) preoperatively, and 77.9 (±27.63 SD) postoperatively. All patients, underwent surgical treatment, with 31.7% undergoing STR and 68.3% GTR. The majority of the patients in this study were treated with the Stupp protocol (32; 76.2%), whereas two patients were treated with radiotherapy alone (4.8%) and eight patients did not receive any further treatment (19.0%). The *IDH1* status was assessed in 88.1% of the included patients, demonstrating a 7.1% *IDH1* mutation rate, which is within the published range [36,37].

Overall, the patients’ characteristics reflect a balanced study cohort, with a high percentage of GTR and a representative KPS.

### 2.2. Overall Survival, Sex, Age and Resection Association

**Figure 1:**
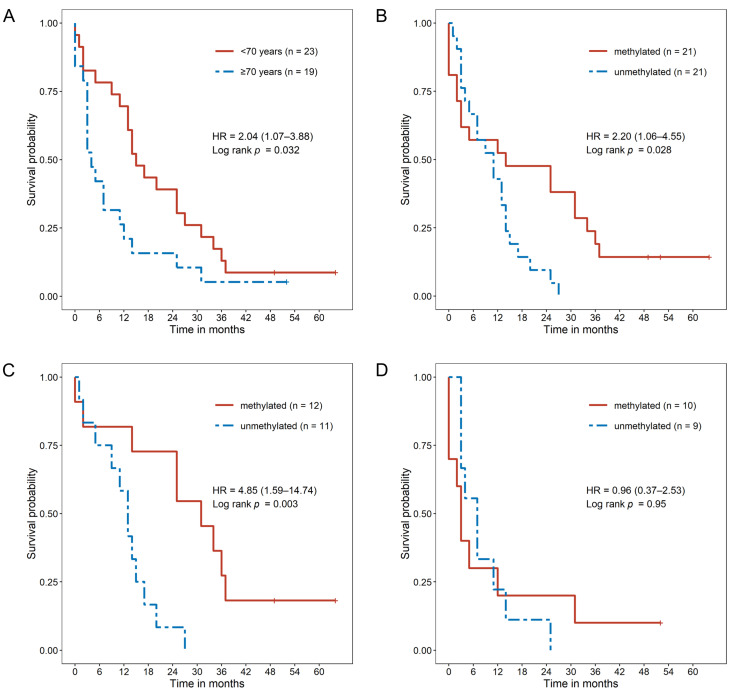
Kaplan–Meier estimates of overall survival (OS) in patients with glioblastoma WHO grade IV. (**A**) OS of patients stratified by age at diagnosis younger than 70 years and older than 70 years. (**B**) OS of patients stratified by *MGMT* promoter methylation status. (**C**) OS in patients younger than 70 years, comparing *MGMT* promoter methylation. (**D**) OS in patients older than 70 years, comparing *MGMT* promoter methylation. The hazard ratio (HR) and the 95% confidence interval in are stated in brackets. Statistical significance between the groups was evaluated using a log-rank test.

The median OS for the entire GBM patient cohort was 11.5 months (95% CI: 5.0–17.0). Stratifying by age, patients <70 years revealed a longer median OS, of 15 months, compared to 4 months for patients ≥70 years (*p* = 0.032) (Figure 1A).

A sex-related difference in OS was not detected (Figure S1), with a median OS for females of 14.5 months (95% CI: 3.0–34.0) compared to 8 months for males (95% CI: 4.0–17.0) (*p* = 0.37) in the total cohort. Within the subgroup of patients with GTR an OS advantage compared to patients with STR (median OS of 13 and 4 months, respectively; *p* = 0.3) was observed (Figure S2), although not reaching statistical significance. For PFS, neither age-related nor a sex-related effects were determined (*p* = 0.460 and *p* = 0.180, respectively). However, GTR compared to STR demonstrated a benefit for PFS (median PFS of 214 and 102 days, respectively, *p* = 0.003). The results are summarized in Table 2.

In summary, the data underline the beneficial influence on OS in younger patients with GBM and, in general, patients with GTR.

### 2.3. MGMT Promoter Methylation Causes a Survival Benefit in Younger GBM Patients

In total, *MGMT* promoter methylation (*MGMT*^met+^) was present in 50% of the patients (representative pyrograms are depicted in Figure S3). Patients with unmethylated *MGMT* promoter (*MGMT*^met−^) did show a slight advantage during the first 6 months after surgery (Figure 1B). This slight advantage could likely be explained by a resection effect, since 76.2% of patients with *MGMT*^met−^ underwent GTR compared to 60.0% GTR in the *MGMT*^met+^ group. However, the median OS was observed to be longer in patients with *MGMT*^met+^ compared to patients with *MGMT*^met−^, at 14 and 11 months, respectively, (*p* = 0.028) (Figure 1B). Stratification of *MGMT* methylation status and age revealed a statistically significant longer OS for younger patients compared to elderly patients. Patients <70 years with *MGMT*^met+^ (*n* = 11) showed a median OS of 31 months compared to the *MGMT*^met−^ subgroup (*n* = 12), with a median OS of 13 months (*p* = 0.003) (Figure 1C). In the ≥70 years subgroup of patients, patients with *MGMT*^met−^ (*n* = 9) and patients with *MGMT*^met+^ (*n* = 10) revealed a similar OS (Figure 1D). The univariate Cox proportional hazard model revealed an increased risk for patients <70 years and an unmethylated MGMT promoter (hazard ratio (HR): 4.85 (95% CI: 1.59–14.74), *p* = 0.005) compared to the patients with *MGMT*^met+^ (Table 3). STR represented a risk for patients ≥70 years (HR: 4.05 (95% CI: 1.11–14.79), *p* = 0.035) compared to GTR. Analyses of PFS demonstrated no survival benefit for any subgroup of GBM patients, independent of age and *MGMT* promoter methylation status.

Overall, the results showed an OS advantage for younger patients with *MGMT*^met+^, while the *MGMT*^met−^ showed a concomitant risk for tumor-related death.

### 2.4. MGMT Promoter Methylation Does Not Correlate with MGMT Protein Expression

**Figure 2:**
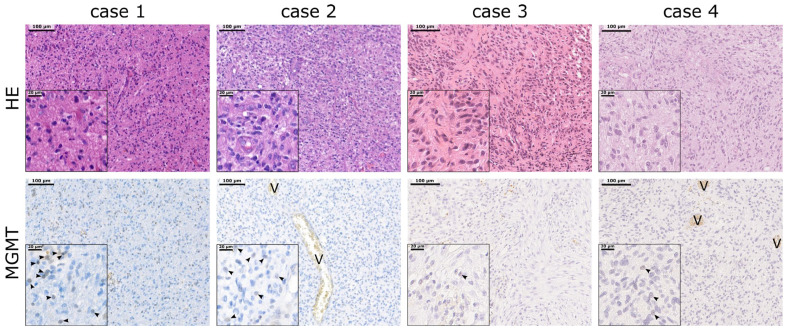
Representative images of the hematoxylin/eosin (HE) staining and the immunohistochemical staining for MGMT protein. Case 1 represents *MGMT*^met+^/MGMT^high^, case 2 *MGMT*^met−^/MGMT^high^, case 3 *MGMT*^met+^/MGMT^low^ and case 4 *MGMT*^met−^/MGMT^low^. Images were taken using the Pannoramic Scan software v1.15.0.57 of a 3D Histech Scanner, and exemplary areas were determined via the Panoramic Viewer software v2.4.0.53492. Arrow heads indicate MGMT-positive cells. V = vessel; scale bar = 100 µm; insert scale bar = 20 µm.

Classification by IHC revealed high MGMT protein expression for 26% of patients (MGMT^high^), while in 74% of patients, low MGMT protein expression (MGMT^low^) could be detected (exemplary images are shown in Figure 2). For both groups, the MGMT^high^ and MGMT^low^ group, comparable percentages of *MGMT* promoter methylation, 45.5% (*n* = 5/11) and 52% (*n* = 16/31), respectively, were determined (Table 2). No correlation could be monitored between *MGMT* promoter methylation and MGMT protein expression (phi = 0.054). Both subgroups with an unmethylated *MGMT* promoter, *MGMT*^met−^/MGMT^low^ and *MGMT*^met−^/MGMT^high^, had a higher tumor-related death probability irrespective of the MGMT protein expression (HR = 2.11 (95% CI: 0.92–4.83), *p* = 0.078 and HR = 3.78 (95% CI: 1.29–11.09), *p* = 0.015, respectively) compared to the *MGMT*^met+^/MGMT^low^ patients.

Association analyses of the different subgroups and the PFS revealed no statistical differences. However, a trend could be monitored, whereby the *MGMT*^met+^/MGMT*^low^* patients (*n* = 12) showed the longest PFS with a median of 281 days compared to all other subgroups. The *MGMT*^met−^/MGMT^low^ patients (*n* = 14), demonstrated a median PFS of 149 days. In both subgroups expressing MGMT protein, a decreased median PFS was observed (*MGMT*^met−^/MGMT^high^, *n* = 4, median PFS 211 days and *MGMT*^met+^/MGMT^high^, *n* = 4, median PFS 118 days), albeit the sub-cohorts encompassed low numbers of patients.

To summarize, epigenetic silencing of *MGMT* by promoter methylation does not correlate with protein expression of MGMT. Although methylated, a sub-cohort of patients showed a clear protein expression pattern (*MGMT*^met+^/MGMT^high^), which might indicate that the epigenetic silencing must not result in a complete downregulation of protein expression. Furthermore, our data suggest a tendency for a decreased median PFS in the MGMT^high^ sub-cohort.

### 2.5. MGMT Promoter Methylation Status as a Prognostic Factor Dependent on Patient Age

A multivariate Cox proportional hazard model, including sex, *MGMT* promoter methylation status, MGMT protein expression status and type of resection, was performed and is summarized in Table 4. In the entire cohort, patients with *MGMT*^met−^ showed an increased associated risk for a reduced OS (HR: 2.69 (95% CI: 1.19–6.07), *p* = 0.017). Patients <70 years indicated a more prognosticating effect of the *MGMT* promoter methylation status (HR: 6.14 (95% CI: 1.78–21.21), *p* = 0.004). For STR, the entire cohort revealed an increased risk of death, although not statistically significant. In patients ≥70 years, a statistically significant increased risk was observed (HR: 5.74 (95% CI: 1.43–23.05), *p* = 0.012).

The results of our analyses underline the importance of a GTR, with a focus on the ≥70 years subgroup of patients compared to STR and evaluation of *MGMT* status in patients <70 years as prognostic factors.

### 2.6. Stable Expression of MMR Proteins Independent of MGMT Status

**Figure 3:**
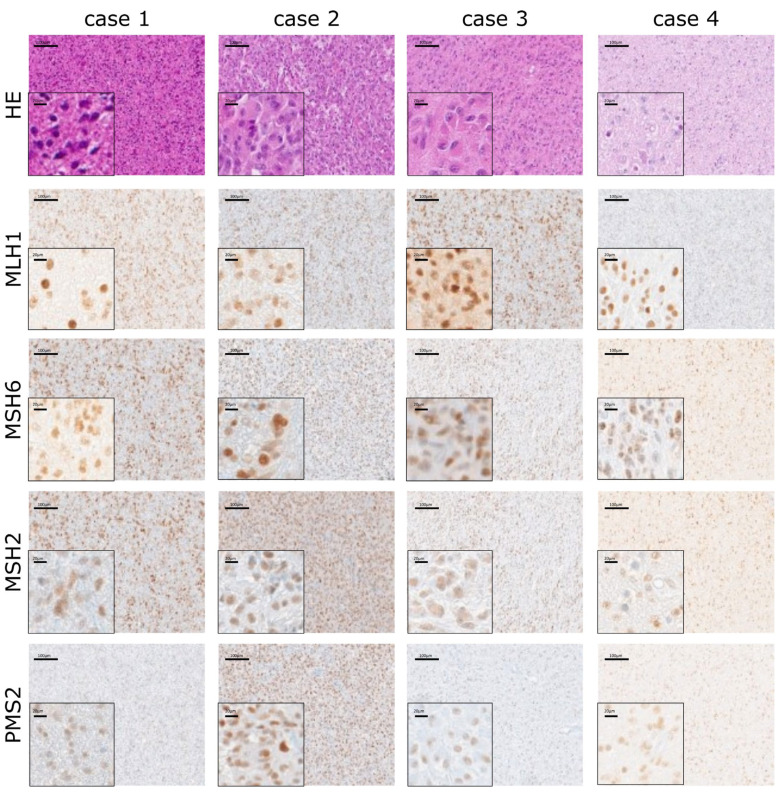
Representative images of the hematoxylin/eosin (HE) staining and the immunohistochemical staining for the mismatch repair system (MMR). All four cases show a positive MMR signal. Case 1 represents *MGMT*^met+^/MMGT^high^, case 2 *MGMT*^met−^/MGMT^high^, case 3 *MGMT*^met+^/MGMT^low^ and case 4 *MGMT*^met−^/MGMT^low^. Brownish color indicates a positive MMR staining with diaminobenzidine. Images were taken using a 3D Histech Scanner, and exemplary areas were determined via the Panoramic Viewer software. Scale bar = 100 µm.

Evaluating the expression results of proteins related to the MMR system, including MLH1, MSH2, MSH6 and PMS2 by IHC, revealed a stable expression in all GBM patients of the tested cohort. MMR protein expression in tumor cells was present with a score of 2 to 3, independent of MGMT status (promoter methylation and protein expression), sex, age, *IDH1* mutation status or treatment (Figure 3).

## 3. Discussion

Prognostic factors are of particular interest in brain tumors. The prognostic value of *MGMT* promoter methylation in newly diagnosed, as well as recurrent GBM is confirmed by a plethora of studies [38]. In our study, we could demonstrate that the *MGMT* promoter methylation status and the combination with MGMT protein expression are of prognostic value in newly diagnosed GBM treated with standard therapy. In particular, the prognostic value of MGMT on epigenetic and protein level is associated with age in GBM patients. Further, the observed effects on OS and PFS seem to be independent from the MMR system. As demonstrated in this study, all tested tumor samples expressed MLH1, MSH2, MSH6 and PMS2 in normal and tumor cells to a high level (both, score and intensity), and there was no correlation with any patient characteristics. Therefore, the evaluation of our data suggests that analyzing protein expression of the MMR system has no impact as a prognostic factor for GBM. Epigenetic silencing of *MGMT* or MGMT protein expression does not influence the expression of MMR.

Generally, the median OS of the entire cohort of this study is comparable to other studies investigating patients with GBM [39,40]. However, other studies show a longer median OS [4,41,42]. This difference is likely due to a higher number of included elderly patients in our study. This reflects published data including elderly individuals [43].

Fifty-two percent of the GBM tested showed *MGMT* promoter methylation resulting in an age-related survival benefit in favor of the <70 year sub-cohort. The relationship between MGMT protein expression and *MGMT* silencing by epigenetic regulation, like hypermethylation of the promoter region, is still controversially discussed. Several studies showed that MGMT protein expression has no clear effect on the clinical outcome of patients [44,45,46]. However, in our study, a subgroup of patients with a low MGMT protein expression and *MGMT* promoter methylation demonstrated a benefit in median OS compared to patients expressing the MGMT protein in tumor cells. Interestingly, even the subgroup with an unmethylated *MGMT* promoter, yet with low MGMT protein expression, revealed a better survival compared to those patients expressing the MGMT protein. The results of both of these defined subgroups are in line with findings of Lalezari et al. [47]. It was indicated that low protein expression alone was inadequate for a better outcome. Interestingly, there is a sub-cohort of *MGMT*-promoter-methylated patients with MGMT protein expression showing a worse median OS. This outcome might be due to a partial escape of the epigenetic silencing of the MGMT protein and an efficient DNA repair via the functional MMR or other DDR systems, such as BER, in the TMZ-treated tumor cells [47,48,49].

Overall, it can be concluded, that a partial correlation of an effective *MGMT* silencing via epigenetic alterations is present in a portion of patients with GBM, while in others a “mosaic-like” pattern, such as unmethylated *MGMT* promoter and low protein expression or methylated *MGMT* promoter and present MGMT protein expression, exist.

Evaluation of our data suggests that analyzing protein expression of the MMR system has no impact as a prognostic factor for GBM. Epigenetic silencing of *MGMT* or MGMT protein expression does not influence the expression of MMR. However, mutations or hypermethylation of the MMR proteins, such as MLH1, a dynamic expression pattern or the involvement of other DDR pathways [48] upon/after TMZ treatment, remain to be clarified in future studies.

Besides molecular factors, the extent of neurosurgical intervention has prognostic value on the survival in GBM [50,51,52]. The results of this study show that GTR of the tumor results in an OS and PFS advantage, whereas STR is associated with decreased survival probabilities dependent on age. This is in line with published data [40,53,54]. Although only elderly patients, ≥65-year-old patients were included in the study by Bruno et al., and a clear advantage of GTR compared to STR was observed for OS and PFS [53]. Further study performed by Almenawer et al., showed an OS of 8.68 and 14.04 months for STR and GTR, respectively, and a PFS of 4.31 months for STR and 7.03 months for GTR [54]. These findings underline the importance of surgery in the neuro-oncological management.

The strengths of the study are (i) exclusively treatment-naïve GBM grade IV patients, (ii) with inclusion of MGMT protein expression with the evaluation of the nucleus and cytoplasmic distribution, the cytoplasmic localization could indicate the trafficking of MGMT loaded with methyl towards proteasome for degradation, and (iii) balanced groups for age, MGMT promoter methylation status and surgical resection type. The major limitation of our study is that stratifying into the groups of interest resulted in small sample sizes in the respective subgroups, thus, solely large effects could be observed.

## 4. Materials and Methods

### 4.1. Patient Selection

The implementation of this study is subject to the ethical votes (AN5220 329/4.4, AN2014-0068 334/424) of the Medical University of Innsbruck. Only patients with writ-ten informed consent were included in the study. Tissue samples of diagnosed GBM patients were collected from 2015 to 2018. The following inclusion criteria were applied: (i) patients diagnosed with GBM WHO grade IV, (ii) absence of other concurrent tumors, (iii) treatment-naïve patients, and (iv) availability of tumor tissue for analyses. Medical history, including age, sex, KPS, surgical resection and therapeutic intervention, were documented and evaluated. OS and PFS were calculated from the date of surgery. Surgical re-section was performed via craniotomy, depending on tumor location and preoperative MRI. The extent of resection was measured on postoperative contrast-enhanced T1 MRI (within 48 h after resection). The extent of the resection was determined as GTR (≥98% tumor removal) and STR (90 to 98% tumor removal).

### 4.2. Tissue Sampling

Surgical specimens were formalin-fixed and, after macroscopic evaluation by a trained pathologist, paraffin-embedded (FFPE). After standard hematoxylin/eosin staining (Diapath, Sanova, Vienna, Austria), diagnosis was performed according to the WHO classification (2016 version) [55] by experienced neuropathologists, at the Medical University of Innsbruck and at the Institute of Neuropathology and Neurochemistry, Medical University of Vienna.

### 4.3. Methylation Analysis

Following the histopathological evaluation by an experienced pathologist, three to five 5 µm thick slices of the FFPE material were used for further DNA isolation. Genomic DNA was isolated using the EZ1 DNA investigator kit or QIAamp DNA FFPE Tissue Kit, and bisulfite conversion was performed using the EpiTect Fast FFPE bisulfite Kit (all Kits from Qiagen, Hilden, Germany), according to manufacturer’s recommendation. For pyrosequencing, the Pyro Mark Therascreen MGMT Kit (Qiagen, Hilden, Germany) was used to assess four CpG sites. In total, 50 ng of bisulfite-converted DNA was used for amplification PCR. All analyses were performed as duplicates. As sequencing controls, an unmethylated and a methylated control DNA were processed in parallel. The CpG sites were located in exon 1 of the human MGMT gene (CpG 76–79). A mean methylation score of <8% was classified as unmethylated, as previously published [56,57].

### 4.4. Immunohistochemical Analysis

For the 42 cases, immunohistochemistry was performed on 2 µm thick slices of FFPE material. Following deparaffination of the slices, MMR protein staining was performed using the OptiView DAB IHC Detection Kit or the Ventana system, as recommended by the manufacturer (Roche, Basel, Switzerland). Immunohistochemical staining of the MGMT protein was performed manually on FFPE sections. For antigen retrieval, a heat-induced epitope retrieval was performed in a water bath at 98.5 °C for 40 min, in a citrate buffer (pH 6.0). For detection, the Dako REAL EnVision Detection System (Agilent, Santa Clara, CA, USA) and peroxidase/DAB+/rabbit/mouse was used. The following antibodies and dilutions were used: anti-MGMT antibody, clone MT3.1 (Merck Millipore, Burlington, MA, USA), 1:100 in blocking solution; MLH1 (M1) (Roche Cell Marque, Basel, Switzerland), 1.4 µg/mL pre-diluted; MSH2 (G219-1129) (Roche Cell Marque, Basel, Switzerland), 3.62 mg/mL pre-diluted; MSH6 (BC/44) (Biocare Medical, Pacheco, CA, USA), 1:50 in sCC1; PMS2 (EPR3947) (Roche Cell Marque, Basel, Switzerland), 14.37 µg/mL pre-diluted; IDH1 R132H (clone H09, Dianova, Hamburg, Germany) 1:30. The intensity of the immunohistochemical staining was scored from 0 to 3, representing very weak, weak, moderate and strong staining, respectively. The density of MMR and MGMT protein expression was scored in percent of the tumor area. The MGMT expression scores were assigned to low (scores: 0 and 1) and high (scores: 2 and 3) expression. The expression patterns of MGMT, MMR and IDH1 were evaluated by at least two independent researchers (J.H., S.S., R.H. and A.M.B.-T.), blinded to MGMT promoter methylation status and further clinical information.

### 4.5. Statistics

Correlation analyses for dichotomous data were assessed using the phi coefficient. Survival probabilities were assessed for OS and PFS. Kaplan–Meier curves were compared using the log-rank test. Univariate and multivariate analyses were carried out using Cox proportional hazards models. A *p*-value of <0.05 was considered as statistically significant. All statistical analyses were performed using R version 4.0.3.

## 5. Conclusions

Summarizing the results of this study, determination of *MGMT* promoter methylation status and concomitant MGMT protein expression status have an additional prognostic value, whereby the former is particularly of prognostic value in patients younger than 70 years. The analysis of the MMR system by IHC could not demonstrate any diagnostic or prognostic relevance in order to improve the treatment of GBM patients. Further analyses are needed in the future to define the role of the epigenetic *MGMT* silencing regulation and its effectiveness and to strengthen its application as a prognostic and predictive molecular marker in GBM.

## Figures and Tables

**Table 1:** Characteristics of patients with glioblastoma WHO grade IV. The characteristics are listed for all patients (All), patients younger than 70 years of age (<70 years) and patients with an age of 70 years or more (≥70 years).

Characteristic	All	<70 Years	≥70 Years
Patients, *n* (%)	42	23 (54.8)	19 (45.2)
Sex			
Female, *n* (%)	16 (38.1)	12 (52.2)	4 (21.1)
Male, *n* (%)	26 (61.9)	11 (47.8)	15 (78.9)
Age at surgery, years, median (IQR)	67.0 (58.0–74.8)	58.0 (54.0–63.0)	75.0 (72.5–78.0)
*IDH1* status			
*IDH1* wildtype, *n* (%)	34 (81.0)	1 (4.3)	2 (10.5)
*IDH1* mutated, *n* (%)	3 (7.1)	18 (78.3)	16 (84.2)
*IDH1* NOS, *n* (%)	5 (11.9)	4 (17.4)	1 (5.3)
KPS			
Preoperative KPS, mean (±SD)	86.3 (±15.45)	89.1 (±9.71)	83.2 (±20.0)
Postoperative KPS, mean (±SD)	77.9 (±27.63)	82.2(±26.45)	72.6 (±28.8)
Extent of surgery ^§^			
Subtotal resection, *n* (%)	13 (31.7)	7 (69.6)	12 (66.7)
Gross total resection, *n* (%)	28 (68.3)	16 (30.4)	6 (33.3)
First-line therapy			
Stupp protocol, *n* (%)	32 (76.2)	21 (95.5)	11 (57.9)
Radiotherapy, *n* (%)	8 (19.0)	2 (4.5)	6 (31.6)
No further treatment, *n* (%)	2 (4.8)	0 (0.0)	2 (10.5)

*IDH1*—isocitrate dehydrogenase 1; KPS—Karnofsky performance score; NOS—not otherwise specified; SD—standard deviation; IQR—interquartile range; ^§^ Data were not evaluable for one patient.

**Table 2:** Overall survival (OS) and progression-free survival (PFS) in patients with glioblastoma WHO grade IV. The data are shown for all patients (All), patients younger than 70 years of age (<70 years) and patients with an age of 70 years or more (≥70 years). The median OS and PFS are given in months and days after surgery, respectively.

	All				<70 Years				≥70 Years			
Parameter	*n*	OS	*n*	PFS	*n*	OS	*n*	PFS	*n*	OS	*n*	PFS
Total cohort	42	11.5	34	116.5	23	15.0	20	125	19	4.0	14	214
Sex												
Female	16	14.5	13	119	12	17.5	10	114	4	1.5	3	294
Male	26	8.0	21	190	11	13.0	10	130	15	5.0	11	214
Age at surgery												
<70 years	23	15.0	20	125								
≥70 years	19	4.0	14	214								
*MGMT* methylation status												
*MGMT*^met+^	21	14.0	16	134	11	31.0	9	125	10	3.0	7	294
*MGMT*^met−^	21	11.0	18	190	12	13.0	11	125	9	7.0	7	214
MGMT protein expression												
MGMT^low^	31	13.0	26	190	18	15.5	16	125	13	4.0	10	214
MGMT^high^	11	7.0	8	134	5	15.0	4	118	6	5.0	4	- *
MGMT combined												
*MGMT*^met+^/MGMT^low^	16	19.5	12	281	8	28.0	7	125	8	2.5	5	294
*MGMT*^met+^/MGMT^high^	5	12.0	4	118	4	17.5	2	118	2	7.5	2	102
*MGMT*^met−^/MGMT^low^	15	13.0	14	149	10	13.0	9	125	5	11.0	5	214
*MGMT*^met−^/MGMT^high^	6	5.0	4	211	1	15.0	2	124	4	4.0	2	- *
Extent of surgery ^§^												
Subtotal resection	13	4	11	102	7	17.0	14	172	6	3	5	294
Gross total resection	28	13	23	214	16	14.5	6	70	12	9	9	- *

MGMT—O6-metylguanin DNA methyltransferase; * Patients censored before half of the patients progressed.; ^§^ Data were not evaluable for one patient.

**Table 3:** Univariate Cox proportional hazard model of OS in patients with glioblastoma WHO grade IV. The data are shown for all patients (All), patients younger than 70 years of age (<70 years) and patients with an age of 70 years or more (≥70 years). References in the model were female sex, methylated *MGMT* promoter, low MGMT protein expression, gross total resection and a Karnofsky performance score below 70. The hazard ratio (HR), the 95% confidence interval (CI) and the corresponding *p*-value are listed.

	All		<70 Years		≥70 Years	
Parameter	HR (95% CI)	*p*	HR (95% CI)	*p*	HR (95% CI)	*p*
Sex (male)	1.34 (0.70–2.58)	0.379	1.24 (0.52–2.94)	0.623	0.75 (0.24–2.36)	0.620
Unmethylated *MGMT* promoter	2.20 (1.06–4.55)	0.034	4.85 (1.59–14.74)	0.005	0.96 (0.37–2.53)	0.941
Expressed MGMT protein	1.48 (0.73–2.99)	0.278	1.19 (0.43–3.31)	0.743	1.40 (0.49–3.97)	0.531
Subtotal resection	1.41 (0.72–2.77)	0.317	1.03 (0.41–2.57)	0.950	4.05 (1.11–14.79)	0.035
KPS pre-surgery (score > 70)	0.80 (0.31–2.06)	0.647	1.99 (0.26–15.20)	0.508	0.78 (0.25–2.42)	0.666

MGMT—O6-methylguanine DNA methyltransferase ; KPS—Karnofsky performance score.

**Table 4:** Multivariate Cox proportional hazard model of OS in patients with GBM. The data are shown for all patients (All), patients younger than 70 years of age (<70 years) and patients with an age of 70 years or more (≥70 years). References in the model were female sex, methylated *MGMT* promoter, low MGMT protein expression and gross total resection. The hazard ratio (HR), the 95% confidence interval (CI) and the corresponding *p*-value are listed.

	All		<70 Years		≥70 Years	
Parameter	HR (95% CI)	*p*	HR (95% CI)	*p*	HR (95% CI)	*p*
Sex (male)	1.36 (0.65–2.87)	0.415	1.00 (0.39–2.51)	0.993	0.70 (0.15–3.23)	0.652
Unmethylated *MGMT* promoter	2.69 (1.19–6.07)	0.017	6.14 (1.78–21.21)	0.004	1.20 (0.39–3.72)	0.753
Expressed MGMT protein	1.67 (0.81–3.44)	0.165	1.53 (0.53–4.43)	0.438	2.56 (0.79–8.32)	0.117
Subtotal resection	1.92 (0.94–3.93)	0.074	1.60 (0.60–4.29)	0.350	5.74 (1.43–23.05)	0.012

MGMT—O6-methylguanine DNA methyltransferase.

## Data Availability

The data presented in this study are available on request from the corresponding author. The data are not publicly available due to ethical restrictions.

## Supplementary Materials

The following supporting information can be downloaded at: https://www.mdpi.com/article/10.3390/ijms24076184/s1.

## References

[1] Abd-Elghany A.A., Naji A.A., Alonazi B., Aldosary H., Alsufayan M.A., Alnasser M., Mohammad E.A., Mahmoud M.Z. (2019). Radiological Characteristics of Glioblastoma Multiforme Using CT and MRI Examination. J. Radiat. Res. Appl. Sci..

[2] Magazzù A., Marcuello C. (2023). Investigation of Soft Matter Nanomechanics by Atomic Force Microscopy and Optical Tweezers: A Comprehensive Review. Nanomaterials.

[3] Tsitlakidis A., Tsingotjidou A.S., Kritis A., Cheva A., Selviaridis P., Aifantis E.C., Foroglou N. (2021). Atomic Force Microscope Nanoindentation Analysis of Diffuse Astrocytic Tumor Elasticity: Relation with Tumor Histopathology. Cancers.

[4] Stupp R., Hegi M.E., Mason W.P., van den Bent M.J., Taphoorn M.J.B., Janzer R.C., Ludwin S.K., Allgeier A., Fisher B., Belanger K. (2009). Effects of Radiotherapy with Concomitant and Adjuvant Temozolomide versus Radiotherapy Alone on Survival in Glioblastoma in a Randomised Phase III Study: 5-Year Analysis of the EORTC-NCIC Trial. Lancet Oncol..

[5] Hegi M.E., Liu L., Herman J.G., Stupp R., Wick W., Weller M., Mehta M.P., Gilbert M.R. (2008). Correlation of O6-Methylguanine Methyltransferase (MGMT) Promoter Methylation with Clinical Outcomes in Glioblastoma and Clinical Strategies to Modulate MGMT Activity. J. Clin. Oncol..

[6] Hegi M.E., Diserens A.-C., Gorlia T., Hamou M.-F., de Tribolet N., Weller M., Kros J.M., Hainfellner J.A., Mason W., Mariani L. (2005). MGMT Gene Silencing and Benefit from Temozolomide in Glioblastoma. N. Engl. J. Med..

[7] Esteller M., Herman J.G. (2004). Generating Mutations but Providing Chemosensitivity: The Role of O6-Methylguanine DNA Methyltransferase in Human Cancer. Oncogene.

[8] Spiegl-Kreinecker S., Pirker C., Filipits M., Lötsch D., Buchroithner J., Pichler J., Silye R., Weis S., Micksche M., Fischer J. (2010). O6-Methylguanine DNA Methyltransferase Protein Expression in Tumor Cells Predicts Outcome of Temozolomide Therapy in Glioblastoma Patients. Neuro Oncol..

[9] Ostrom Q.T., Bauchet L., Davis F.G., Deltour I., Fisher J.L., Langer C.E., Pekmezci M., Schwartzbaum J.A., Turner M.C., Walsh K.M. (2014). The Epidemiology of Glioma in Adults: A “State of the Science” Review. Neuro Oncol..

[10] Zhang K., Wang X., Zhou B., Zhang L. (2013). The Prognostic Value of MGMT Promoter Methylation in Glioblastoma Multiforme: A Meta-Analysis. Fam. Cancer.

[11] Hegi M.E., Diserens A.-C., Godard S., Dietrich P.-Y., Regli L., Ostermann S., Otten P., Van Melle G., de Tribolet N., Stupp R. (2004). Clinical Trial Substantiates the Predictive Value of O-6-Methylguanine-DNA Methyltransferase Promoter Methylation in Glioblastoma Patients Treated with Temozolomide. Clin. Cancer Res..

[12] Esteller M., Toyota M., Sanchez-Cespedes M., Capella G., Peinado M.A., Watkins D.N., Issa J.P., Sidransky D., Baylin S.B., Herman J.G. (2000). Inactivation of the DNA Repair Gene O6-Methylguanine-DNA Methyltransferase by Promoter Hypermethylation Is Associated with G to A Mutations in K-Ras in Colorectal Tumorigenesis. Cancer Res..

[13] Uno M., Oba-Shinjo S.M., Camargo A.A., Moura R.P., de Aguiar P.H., Cabrera H.N., Begnami M., Rosemberg S., Teixeira M.J., Marie S.K.N. (2011). Correlation of MGMT Promoter Methylation Status with Gene and Protein Expression Levels in Glioblastoma. Clinics.

[14] Stupp R., Mason W.P., van den Bent M.J., Weller M., Fisher B., Taphoorn M.J.B., Belanger K., Brandes A.A., Marosi C., Bogdahn U. (2005). Radiotherapy plus Concomitant and Adjuvant Temozolomide for Glioblastoma. N. Engl. J. Med..

[15] Stupp R., Taillibert S., Kanner A.A., Kesari S., Steinberg D.M., Toms S.A., Taylor L.P., Lieberman F., Silvani A., Fink K.L. (2015). Maintenance Therapy with Tumor-Treating Fields plus Temozolomide vs. Temozolomide Alone for Glioblastoma: A Randomized Clinical Trial. JAMA.

[16] Wick W., Platten M., Meisner C., Felsberg J., Tabatabai G., Simon M., Nikkhah G., Papsdorf K., Steinbach J.P., Sabel M. (2012). Temozolomide Chemotherapy Alone versus Radiotherapy Alone for Malignant Astrocytoma in the Elderly: The NOA-08 Randomised, Phase 3 Trial. Lancet Oncol..

[17] Malmström A., Grønberg B.H., Marosi C., Stupp R., Frappaz D., Schultz H., Abacioglu U., Tavelin B., Lhermitte B., Hegi M.E. (2012). Temozolomide versus Standard 6-Week Radiotherapy versus Hypofractionated Radiotherapy in Patients Older than 60 Years with Glioblastoma: The Nordic Randomised, Phase 3 Trial. Lancet Oncol..

[18] Jiapaer S., Furuta T., Tanaka S., Kitabayashi T., Nakada M. (2018). Potential Strategies Overcoming the Temozolomide Resistance for Glioblastoma. Neurol. Med. Chir..

[19] Li G.-M. (2008). Mechanisms and Functions of DNA Mismatch Repair. Cell Res..

[20] Pegg A.E., Dolan M.E., Moschel R.C. (1995). Structure, Function, and Inhibition of O6-Alkylguanine-DNA Alkyltransferase. Prog. Nucleic Acid Res. Mol. Biol..

[21] Koukourakis G.V., Kouloulias V., Zacharias G., Papadimitriou C., Pantelakos P., Maravelis G., Fotineas A., Beli I., Chaldeopoulos D., Kouvaris J. (2009). Temozolomide with Radiation Therapy in High Grade Brain Gliomas: Pharmaceuticals Considerations and Efficacy; a Review Article. Molecules.

[22] Trivedi R.N., Almeida K.H., Fornsaglio J.L., Schamus S., Sobol R.W. (2005). The Role of Base Excision Repair in the Sensitivity and Resistance to Temozolomide-Mediated Cell Death. Cancer Res..

[23] Helleday T., Petermann E., Lundin C., Hodgson B., Sharma R.A. (2008). DNA Repair Pathways as Targets for Cancer Therapy. Nat. Rev. Cancer.

[24] Maxwell J.A., Johnson S.P., McLendon R.E., Lister D.W., Horne K.S., Rasheed A., Quinn J.A., Ali-Osman F., Friedman A.H., Modrich P.L. (2008). Mismatch Repair Deficiency Does Not Mediate Clinical Resistance to Temozolomide in Malignant Glioma. Clin. Cancer Res..

[25] Perazzoli G., Prados J., Ortiz R., Caba O., Cabeza L., Berdasco M., Gónzalez B., Melguizo C. (2015). Temozolomide Resistance in Glioblastoma Cell Lines: Implication of MGMT, MMR, P-Glycoprotein and CD133 Expression. PLoS ONE.

[26] Quiros S., Roos W.P., Kaina B. (2010). Processing of O6-Methylguanine into DNA Double-Strand Breaks Requires Two Rounds of Replication Whereas Apoptosis Is Also Induced in Subsequent Cell Cycles. Cell Cycle.

[27] Novo N., Romero-Tamayo S., Marcuello C., Boneta S., Blasco-Machin I., Velázquez-Campoy A., Villanueva R., Moreno-Loshuertos R., Lostao A., Medina M. (2022). Beyond a Platform Protein for the Degradosome Assembly: The Apoptosis-Inducing Factor as an Efficient Nuclease Involved in Chromatinolysis. PNAS Nexus.

[28] Artus C., Boujrad H., Bouharrour A., Brunelle M.N., Hoos S., Yuste V.J., Lenormand P., Rousselle J.C., Namane A., England P. (2010). AIF Promotes Chromatinolysis and Caspase-Independent Programmed Necrosis by Interacting with Histone H2AX. EMBO J..

[29] Wu B.-P., Zhang Y.-L., Zhou D.-Y., Gao C.-F., Lai Z.-S. (2000). Microsatellite Instability, MMR Gene Expression and Proliferation Kinetics in Colorectal Cancer with Famillial Predisposition. World J. Gastroenterol..

[30] McConechy M.K., Talhouk A., Li-Chang H.H., Leung S., Huntsman D.G., Gilks C.B., McAlpine J.N. (2015). Detection of DNA Mismatch Repair (MMR) Deficiencies by Immunohistochemistry Can Effectively Diagnose the Microsatellite Instability (MSI) Phenotype in Endometrial Carcinomas. Gynecol. Oncol..

[31] Carethers J.M., Stoffel E.M. (2015). Lynch Syndrome and Lynch Syndrome Mimics: The Growing Complex Landscape of Hereditary Colon Cancer. World J. Gastroenterol..

[32] Cancer Genome Atlas Research Network (2008). Comprehensive Genomic Characterization Defines Human Glioblastoma Genes and Core Pathways. Nature.

[33] Yip S., Miao J., Cahill D.P., Iafrate A.J., Aldape K., Nutt C.L., Louis D.N. (2009). MSH6 Mutations Arise in Glioblastomas during Temozolomide Therapy and Mediate Temozolomide Resistance. Clin. Cancer Res..

[34] Felsberg J., Thon N., Eigenbrod S., Hentschel B., Sabel M.C., Westphal M., Schackert G., Kreth F.W., Pietsch T., Löffler M. (2011). Promoter Methylation and Expression of MGMT and the DNA Mismatch Repair Genes MLH1, MSH2, MSH6 and PMS2 in Paired Primary and Recurrent Glioblastomas. Int. J. Cancer.

[35] Hunter C., Smith R., Cahill D.P., Stephens P., Stevens C., Teague J., Greenman C., Edkins S., Bignell G., Davies H. (2006). A Hypermutation Phenotype and Somatic MSH6 Mutations in Recurrent Human Malignant Gliomas after Alkylator Chemotherapy. Cancer Res..

[36] Parsons D.W., Jones S., Zhang X., Lin J.C.-H., Leary R.J., Angenendt P., Mankoo P., Carter H., Siu I.-M., Gallia G.L. (2008). An Integrated Genomic Analysis of Human Glioblastoma Multiforme. Science.

[37] Huang L.E. (2019). Friend or Foe-IDH1 Mutations in Glioma 10 Years On. Carcinogenesis.

[38] Alnahhas I., LaHaye S., Giglio P., Mardis E., Puduvalli V. (2020). An Evaluation of MGMT Promoter Methylation within the Methylation Subclasses of Glioblastoma. Neuro-Oncol. Adv..

[39] Pinson H., Hallaert G., Van der Meulen J., Dedeurwaerdere F., Vanhauwaert D., Van den Broecke C., Van Dorpe J., Van Roost D., Kalala J.P., Boterberg T. (2020). Weak MGMT Gene Promoter Methylation Confers a Clinically Significant Survival Benefit in Patients with Newly Diagnosed Glioblastoma: A Retrospective Cohort Study. J. Neurooncol..

[40] Sanai N., Polley M.-Y., McDermott M.W., Parsa A.T., Berger M.S. (2011). An Extent of Resection Threshold for Newly Diagnosed Glioblastomas. J. Neurosurg..

[41] Magrowski Ł., Nowicka E., Masri O., Tukiendorf A., Tarnawski R., Miszczyk M. (2021). The Survival Impact of Significant Delays between Surgery and Radiochemotherapy in Glioblastoma Patients: A Retrospective Analysis from a Large Tertiary Center. J. Clin. Neurosci..

[42] Hallaert G., Pinson H., Vanhauwaert D., Van den Broecke C., Van Roost D., Boterberg T., Kalala J.P. (2020). Partial Resection Offers an Overall Survival Benefit over Biopsy in MGMT-Unmethylated IDH-Wildtype Glioblastoma Patients. Surg. Oncol..

[43] Abhinav K., Aquilina K., Gbejuade H., La M., Hopkins K., Iyer V. (2013). A Pilot Study of Glioblastoma Multiforme in Elderly Patients: Treatments, O-6-Methylguanine-DNA Methyltransferase (MGMT) Methylation Status and Survival. Clin. Neurol. Neurosurg..

[44] Cao V.T., Jung T.Y., Jung S., Jin S.G., Moon K.S., Kim I.Y., Kang S.S., Park C.S., Lee K.H., Chae H.J. (2009). The Correlation and Prognostic Significance of MGMT Promoter Methylation and MGMT Protein in Glioblastomas. Neurosurgery.

[45] Rodriguez F.J., Thibodeau S.N., Jenkins R.B., Schowalter K.V., Caron B.L., O’neill B.P., James C.D., David James C., Passe S., Slezak J. (2008). MGMT Immunohistochemical Expression and Promoter Methylation in Human Glioblastoma. Appl. Immunohistochem. Mol. Morphol..

[46] Sonoda Y., Yokosawa M., Saito R., Kanamori M., Yamashita Y., Kumabe T., Watanabe M., Tominaga T. (2010). O(6)-Methylguanine DNA Methyltransferase Determined by Promoter Hypermethylation and Immunohistochemical Expression Is Correlated with Progression-Free Survival in Patients with Glioblastoma. Int. J. Clin. Oncol..

[47] Lalezari S., Chou A.P., Tran A., Solis O.E., Khanlou N., Chen W., Li S., Carrillo J.A., Chowdhury R., Selfridge J. (2013). Combined Analysis of O6-Methylguanine-DNA Methyltransferase Protein Expression and Promoter Methylation Provides Optimized Prognostication of Glioblastoma Outcome. Neuro Oncol..

[48] Gao F., Cui Y., Jiang H., Sui D., Wang Y., Jiang Z., Zhao J., Lin S. (2016). Circulating Tumor Cell Is a Common Property of Brain Glioma and Promotes the Monitoring System. Oncotarget.

[49] Barciszewska A.-M., Gurda D., Głodowicz P., Nowak S., Naskręt-Barciszewska M.Z. (2015). A New Epigenetic Mechanism of Temozolomide Action in Glioma Cells. PLoS ONE.

[50] Zinn P.O., Colen R.R., Kasper E.M., Burkhardt J.-K. (2013). Extent of Resection and Radiotherapy in GBM: A 1973 to 2007 Surveillance, Epidemiology and End Results Analysis of 21,783 Patients. Int. J. Oncol..

[51] Brown T.J., Brennan M.C., Li M., Church E.W., Brandmeir N.J., Rakszawski K.L., Patel A.S., Rizk E.B., Suki D., Sawaya R. (2016). Association of the Extent of Resection With Survival in Glioblastoma: A Systematic Review and Meta-Analysis. JAMA Oncol..

[52] Awad A.-W., Karsy M., Sanai N., Spetzler R., Zhang Y., Xu Y., Mahan M.A. (2017). Impact of Removed Tumor Volume and Location on Patient Outcome in Glioblastoma. J. Neurooncol..

[53] Bruno F., Pellerino A., Pronello E., Palmiero R., Bertero L., Mantovani C., Bianconi A., Melcarne A., Garbossa D., Rudà R. (2022). Elderly Gliobastoma Patients: The Impact of Surgery and Adjuvant Treatments on Survival: A Single Institution Experience. Brain Sci..

[54] Almenawer S.A., Badhiwala J.H., Alhazzani W., Greenspoon J., Farrokhyar F., Yarascavitch B., Algird A., Kachur E., Cenic A., Sharieff W. (2015). Biopsy versus Partial versus Gross Total Resection in Older Patients with High-Grade Glioma: A Systematic Review and Meta-Analysis. Neuro Oncol..

[55] Louis D.N., Perry A., Reifenberger G., von Deimling A., Figarella-Branger D., Cavenee W.K., Ohgaki H., Wiestler O.D., Kleihues P., Ellison D.W. (2016). The 2016 World Health Organization Classification of Tumors of the Central Nervous System: A Summary. Acta Neuropathol..

[56] Reifenberger G., Hentschel B., Felsberg J., Schackert G., Simon M., Schnell O., Westphal M., Wick W., Pietsch T., Loeffler M. (2012). Predictive Impact of MGMT Promoter Methylation in Glioblastoma of the Elderly. Int. J. Cancer.

[57] Preusser M., Berghoff A.S., Manzl C., Filipits M., Weinhäusel A., Pulverer W., Dieckmann K., Widhalm G., Wöhrer A., Knosp E. (2014). Clinical Neuropathology Practice News 1-2014: Pyrosequencing Meets Clinical and Analytical Performance Criteria for Routine Testing of MGMT Promoter Methylation Status in Glioblastoma. Clin. Neuropathol..

