# Supplementary material for: Influence of MMR, MGMT Promotor Methylation and Protein Expression on Overall and Progression-Free Survival in Primary Glioblastoma Patients Treated with Temozolomide
Source: Int J Mol Sci. 2023 Mar 24;24(7):6184. doi: 10.3390/ijms24076184 (PMC10094528; doi:10.3390/ijms24076184)
Supplement: Supplementary file 1 [file ijms-24-06184-s001.zip › ijms-2282855-supplementary.docx]

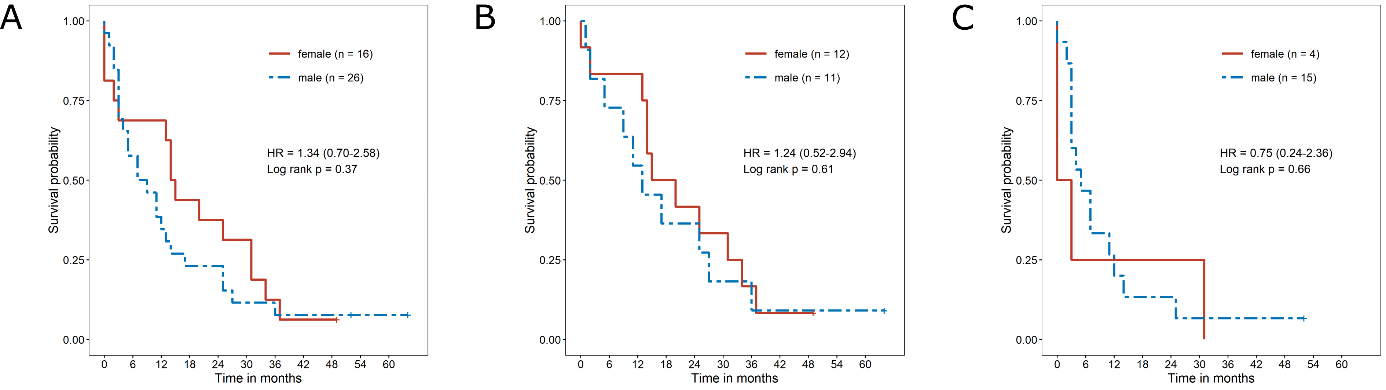


**Figure S1.** Kaplan Meier plots of overall survival (OS) in patients with glioblastoma WHO grade IV stratified by sex. (**A**) OS of all included patients stratified by sex. (**B**) OS of patients in patients younger than 70 years stratified by sex. (**C**) OS in patients older than 70 years stratified by sex. The hazard ratio (HR) and the 95% confidence interval in brackets are stated. Statistical significance between groups was evaluated using log rank test.


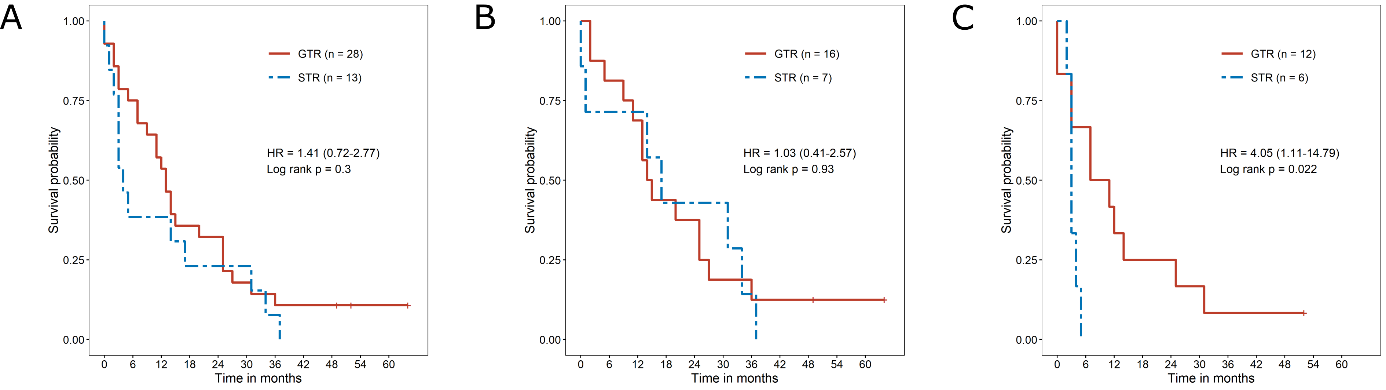


**Figure S2.** Kaplan Meier plots of overall survival (OS) in patients with glioblastoma WHO grade IV stratified by the extent of surgery. (**A**) OS of all included patients stratified by the extent of surgery. (**B**) OS of patients in patients younger than 70 years stratified by the extent of surgery. (**C**) OS in patients older than 70 years stratified by the extent of surgery. The hazard ratio (HR) and the 95% confidence interval in brackets are stated. Statistical significance between groups was evaluated using log rank test.


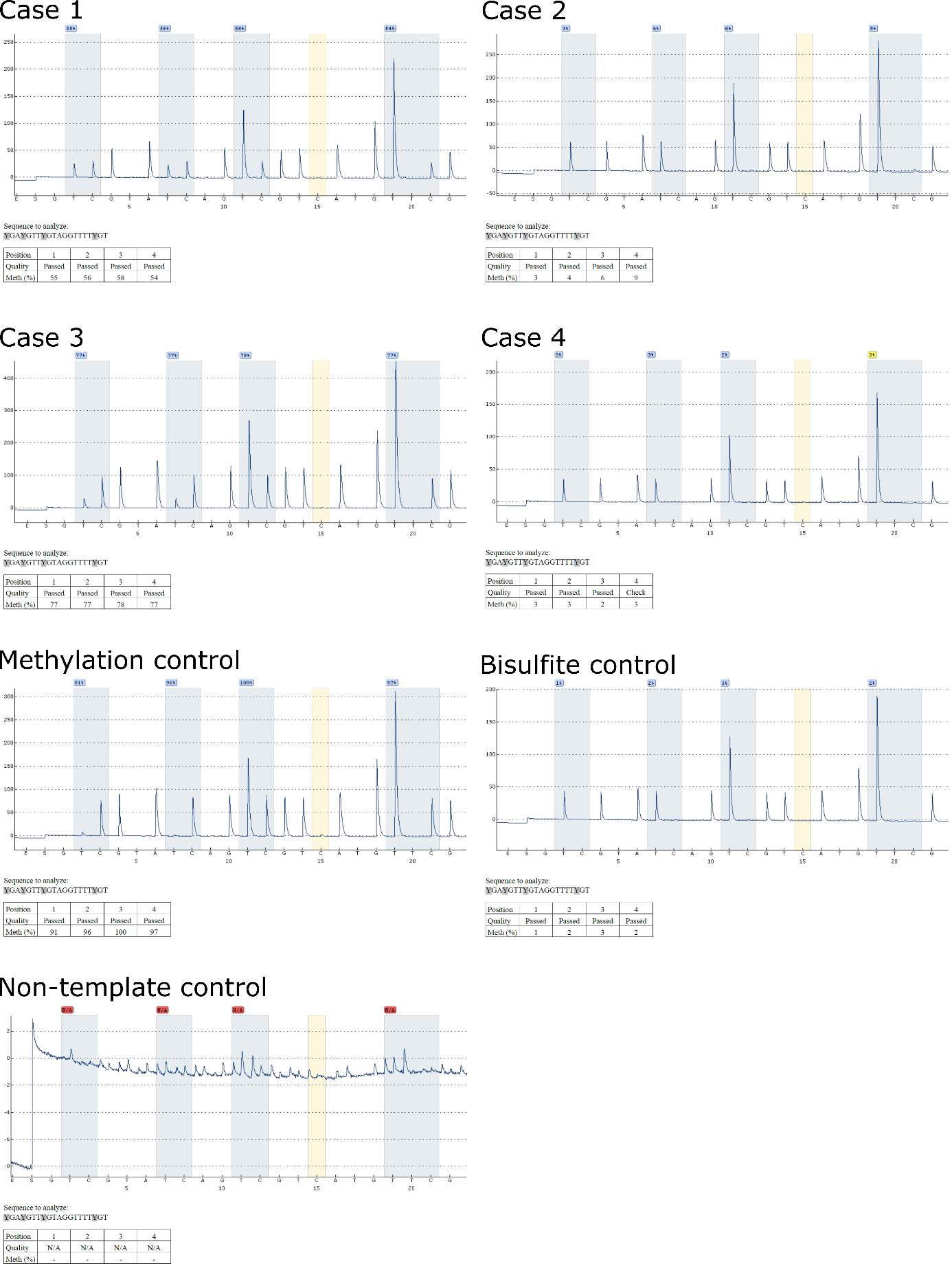


**Figure S3.** Representative pyrograms of cases 1-4 and controls.
